# Intra-amniotic LPS causes acute neuroinflammation in preterm rhesus macaques

**DOI:** 10.1186/s12974-016-0706-4

**Published:** 2016-09-06

**Authors:** Augusto F. Schmidt, Paranthaman S. Kannan, Claire A. Chougnet, Steve C. Danzer, Lisa A. Miller, Alan H. Jobe, Suhas G. Kallapur

**Affiliations:** 1 Division of Neonatology and Pulmonary Biology, Cincinnati Children’s Hospital Medical Center, 3333 Burnet Ave, Cincinnati, OH 45229 USA; 2 Division of Immunobiology, Cincinnati Children’s Hospital Medical Center, Cincinnati, OH USA; 3 Department of Anesthesia, Cincinnati Children’s Hospital Medical Center, Cincinnati, OH USA; 4 California National Primate Research Center and Department of Pediatrics and Cell Biology and Human Anatomy, University of California, Davis, CA USA

**Keywords:** Chorioamnionitis, Prematurity, Brain injury, Periventricular leukomalacia, Cytokines, Microglia, Apoptosis

## Abstract

**Background:**

Chorioamnionitis is associated with an increased risk of brain injury in preterm neonates. Inflammatory changes in brain could underlie this injury. Here, we evaluated whether neuroinflammation is induced by chorioamnionitis in a clinically relevant model.

**Methods:**

Rhesus macaque fetuses were exposed to either intra-amniotic (IA) saline, or IA lipopolysaccharide (LPS) (1 mg) 16 or 48 h prior to delivery at 130 days (85 % of gestation) (*n* = 4–5 animals/group). We measured cytokines in the cerebrospinal fluid (CSF), froze samples from the left brain for molecular analysis, and immersion fixed the right brain hemisphere for immunohistology. We analyzed the messenger RNA (mRNA) levels of the pro-inflammatory cytokines IL-1β, CCL2, TNF-α, IL-6, IL-8, IL-10, and COX-2 in the periventricular white matter (PVWM), cortex, thalamus, hippocampus, and cerebellum by RT-qPCR. Brain injury was assessed by immunohistology for myelin basic protein (MBP), IBA1 (microglial marker), GFAP (astrocyte marker), OLIG2 (oligodendrocyte marker), NeuN (neuronal marker), CD3 (T cells), and CD14 (monocytes). Microglial proliferation was assessed by co-immunostaining for IBA1 and Ki67. Data were analyzed by ANOVA with Tukey’s post-test.

**Results:**

IA LPS increased mRNA expression of pro-inflammatory cytokines in the PVWM, thalamus, and cerebellum, increased IL-6 concentration in the CSF, and increased apoptosis in the periventricular area after 16 h. Microglial proliferation in the white matter was increased 48 h after IA LPS.

**Conclusions:**

LPS-induced chorioamnionitis caused neuroinflammation, microglial proliferation, and periventricular apoptosis in a clinically relevant model of chorioamnionitis in fetal rhesus macaques. These findings identify specific responses in the fetal brain and support the hypothesis that neuroinflammatory changes may mediate the adverse neurodevelopmental outcomes associated with chorioamnionitis.

**Electronic supplementary material:**

The online version of this article (doi:10.1186/s12974-016-0706-4) contains supplementary material, which is available to authorized users.

## Background

Chorioamnionitis is frequently associated with preterm birth [1], and fetal exposure to inflammation is an independent risk factor for brain injury, including intraventricular hemorrhage (IVH), periventricular leukomalacia (PVL), cerebral palsy (CP), and cognitive impairment [2, 3]. In a multicenter prospective study of 3094 infants born before 33 weeks’ gestational age, clinical chorioamnionitis increased the risk of severe intraventricular hemorrhage, with an odds ratio of 1.62 [4]. Histological chorioamnionitis increased the risk of intraventricular hemorrhage, periventricular leukomalacia, and cerebral palsy on neurodevelopmental testing between 30 and 42 months corrected age (odds ratio 2.45) in a recent retrospective study with prospective follow-up including infants born with less than 29 weeks gestational age [5].

Selection of the animal model is a critical variable for research examining chorioamnionitis-induced brain injury. The most commonly used model for inflammation induced brain injury combines hypoxia/ischemia and intraperitoneal lipopolysaccharide (LPS) in term newborn rodents [6]. Neuroinflammation, characterized by microglial activation and damage to the white matter, is consistently observed in this model [7]. Additionally, preterm infants with exposure to histological chorioamnionitis have only modest increases in inflammatory cytokines in the serum and do not have manifestations of systemic inflammatory response [8].

Systemic inflammation can cause an inflammatory response in the fetal brain with microglial activation, upregulation of cytokines, and neuronal apoptosis [9, 10]. In neonatal rats, intra-cerebral injection of IL-1β induces apoptosis and astrogliosis and decreases myelin basic protein (MBP) staining [11]. The IL-1β injection results in long-term neuro-inflammatory responses and motor behavioral deficits, which are attenuated by an IL-1 receptor antagonist [12]. Another cytokine that has been implicated in the pathogenesis of brain injury in preterm infants is IL-6. Increased IL-6 concentrations in the cord blood of preterm newborns are associated with increased risk of white matter damage, germinal matrix hemorrhage, and cystic lesions [13, 14]. Brain injury secondary to diverse insults such as trauma, excitatory injury, and infection is also associated with increased expression of COX-2, but there are no data regarding its role in brain injury associated with chorioamnionitis [15].

Inflammation in human chorioamnionitis is largely localized to the uterus, without overt fetal hypoxia or strong maternal systemic inflammatory responses. In fact, measurements of systemic inflammatory markers such as procalcitonin, CRP and IL-6 in the maternal serum do not identify intrauterine inflammation [16, 17]. In contrast to intraperitoneal or systemic LPS injections, intra-amniotic (IA) injections of pro-inflammatory mediators such as IL-1β or LPS induce a predominantly chorioamniotic inflammation with subtle fetal systemic inflammation, similar to the pathology in humans [18]. In preterm fetal sheep, IL-1β was upregulated in the hippocampus, cortex, and cerebellum 2 days after exposure to IA LPS [19], followed by microglial and astrocyte recruitment, apoptosis, and decreased myelination of the subcortical white matter and hippocampus after 7 days [20]. Nonetheless, although studies in rodents and sheep provide some insight into the human pathology, there are striking differences in neurological development and vulnerability to brain injury between these species and humans.

In humans, generation of oligodendrocytes peaks between 23 and 32 weeks of gestation, while in rodents, peak oligodendrogenesis is delayed until 1 to 3 days after birth. Similarly, peak brain growth and gliogenesis occur between 36 and 40 weeks’ gestation in humans, whereas in rodents, the peak occurs between postnatal days 7 to 10 [21]. In contrast to rodents, where development is delayed relative to humans, sheep develop at a faster rate than humans. In sheep, 50 % of the adult brain weight is achieved at 80 % of gestation, while humans do not reach this milestone until early infancy. Similarly, the internal capsule myelination starts at 70 % of gestation in sheep and not until 40 days postnatally in humans [22]. In contrast, rhesus macaques reach 50 % of adult brain weight at term and peak internal capsule myelination starts at 90 % gestation, closer to human development. Thus, nonhuman primate models, such as rhesus macaques, are especially attractive due to similar ontogeny of the nervous systems [22].

We tested the hypothesis that IA administration of LPS will induce inflammation in the central nervous system in the rhesus macaque.

## Methods

### Animals

The Institutional Animal Care and Use Committee at the University of California Davis approved all animal procedures, which were performed at the California National Primate Research Center (CNPRC), University of California, Davis. Time-mated pregnant rhesus macaques were sedated with ketamine at approximately day 130 of gestation (term is 165 days) for ultrasound exam and IA injection of either LPS (1 mg, *E. coli* O55:B5, Sigma Aldrich, Saint Louis, MO) diluted in 1 mL of sterile saline solution or 1 mL of sterile saline as the control injection. After 16 or 48 h, fetuses were surgically delivered for fetal tissue collection. We collected cerebrospinal fluid (CSF) by occipital puncture. The left brain was dissected and the cortex, periventricular white matter (PVWM), thalamus, hippocampus, and cerebellum were snap frozen for molecular analysis. The right brain was fixed in 10 % formalin. We used four to five animals for each group.

### mRNA quantitation

Total RNA was isolated from frozen brains after homogenization with TRIzol (Invitrogen, Carlsbad, CA) and column purified with RNeasy Universal MiniKit (Qiagen, Valencia, CA) according to the manufacturer’s instructions. Reverse transcription was performed using Verso complementary DNA (cDNA) kit (Thermo Scientific, Waltham, MA) to produce single-strand cDNA. The genes for IL-1β, CCL2, TNF-α, IL-6, IL-8, IL-10, cyclooxygenase-2 (COX-2), and prostaglandin E synthase 2 (PTGES2) were amplified by RT-PCR using the cDNA template and rhesus macaque-specific primers along with Taqman probes (Applied Biosystems, Foster City, CA). The messenger RNA (mRNA) expression for each gene was normalized to the mRNA for the ribosomal protein 18 s as internal standard. Data are expressed as fold increase over the mean control value.

### Cytokine measurements

We measured cytokine concentrations in the CSF by Luminex using nonhuman primate multiplex kits (Millipore, Billerica, MA) according to the manufacturer’s protocol. Concentrations were calculated from standard curves using recombinant proteins and expressed in picograms per milliliter.

### Immunohistochemistry

Sections from formalin fixed tissues in paraffin blocks were deparaffinized and rehydrated before microwave-assisted antigen retrieval in citric acid buffer at pH 6.0. Endogenous peroxidase activity was reduced with CH_3_OH/H_2_O_2_ treatment, and the tissue was blocked with 2 % donkey serum in phosphate buffer saline (PBS). Sections were incubated overnight at 4 °C with the primary antibody diluted in 2 % serum in PBS. We used the following primary antibodies: goat polyclonal Anti-Iba1 (Abcam, Cambridge, MA, cat. #5076, dilution 1:200), rabbit monoclonal Anti-glial fibrillary acidic protein (GFAP) (Abcam, Cambridge, MA, cat. #48050, dilution 1:500), rabbitt monoclonal Anti-Olig2 (Abcam, Cambridge, MA, cat. #109186, dilution 1:500), mouse monoclonal Anti-MBP (Abcam, Cambridge, MA, cat. #62631, dilution 1:500), rabbit monoclonal Anti-Tau (Abcam, Cambridge, MA, cat. #32057, dilution 1:500), mouse monoclonal anti-NeuN (Abcam, Cambridge, MA, cat. #ab104224, dilution 1:200), rabbit polyclonal anti-CD3 (DakoCytomation, Carpinteria, CA, cat. #A0452, dilution 1:100), and mouse monoclonal anti-CD14 (BD Biosciences, San Diego, CA, cat. #557742, dilution 1:100). Sections were then washed and incubated with the appropriate species-specific secondary antibody diluted 1:200 in 2 % serum for 2 h at room temperature. After further washing, antigen/antibody complexes were visualized using a Vectastain ABC peroxidase kit (Vector Laboratories Inc., Burlingame, CA). Antigen detection was enhanced with nickel-diaminobenzidine, followed by incubation with Tris-cobalt. Slides were counterstained with Nuclear Fast Red for photomicroscopy.

### Double-labeling immunofluorescence

To identify proliferating microglia, we performed double-labeling immunofluorescence for Iba-1 and Ki-67. Antigen retrieval and blocking were conducted as described above, followed by overnight incubation with goat polyclonal Anti-Iba1 (Abcam, Cambridge, MA, cat. #5076, dilution 1:100) and rat monoclonal anti-Ki67 (LifeSpan Biosciences, Seattle, WA, cat. #LS-C175347, dilution 1:50). The following day, sections were washed and incubated with anti-goat Alexa Fluor 594 (Life technologies, Carlsbad, CA, cat. #A-11058, dilution 1:200) and anti-rat Alexa Fluor 488 (Life technologies, Carlsbad, CA, cat. #A-21470, dilution 1:200) for 2 h at room temperature, followed by washing and incubation with DAPI (Life technologies, Carlsbad, CA, cat. #D1306, dilution 1:2000) for 15 min at room temperature. Sections were washed and mounted with ProLong Gold (Life technologies, Carlsbad, CA, cat. #P36930). Stained slides were imaged on confocal microscopy for co-localization at ×40x magnification with 1024 × 1024 pixel resolution on a Nikon Eclipse A1RSi inverted microscope (Nikon Instruments Inc, Melville, NY).

### Cell counting

Cells immunostained for CD3 (T cells), CD14 (monocytes), and caspase-3 (apoptotic cells) were counted in six randomly selected fields per animal on photomicrographs (×40) from the periventricular area and counted by a masked investigator. A similar strategy was used to assess the number (Iba1+) and proportion of proliferating microglia (Ki67+/Iba1+) in the white matter. A masked investigator counted the number of cells positive for Iba1, and the number of cells positive for both Iba1 and Ki67 in each determined both the number of Iba1+ cells and the percentage of Iba1+/Ki67+ cells.

### Statistical analysis

Results of the mRNA quantitation and cell counting were analyzed by ANOVA followed by post-testing comparison of each experimental group against the control group with Tukey correction. Statistical analysis was performed on GraphPad Prism version 6.0 for Mac OS X (GraphPad Software, San Diego, CA). Data are presented as means ± standard deviation. Values of *p* < 0.05 were considered significant.

## Results

### Animals

Gestational ages at birth and birth weights were similar among the animals (Table 1). One animal in the LPS 48 h group was a fetal death and was excluded from the study. There was a non-significant trend towards increased cord white blood cell and neutrophil counts after IA LPS. The percentage of band form of neutrophils significantly increased in the LPS 16 h group compared to control (*p* < 0.05).

**Table 1:** Animal data

	*n*	Gestational age (days)	Birth weight (grams)	Sex (M/F)	WBC (10^9^/L)*	Neutrophils (%)	Bands (%)
Control	5	132 ± 2	343 ± 9	3/2	2.0 ± 0.9	9 ± 3	0
LPS 16 h	5	131 ± 1	333 ± 43	2/3	2.0 ± 0.4	14 ± 15	1.5 ± 1*
LPS 48 h	4	130 ± 2	307 ± 17	0/4	3.3 ± 1.7	25 ± 10	1 ± 1

*WBC* white blood cell count

*p < 0.05

### IA LPS increases cytokine expression in the brain

**Fig. 1:**
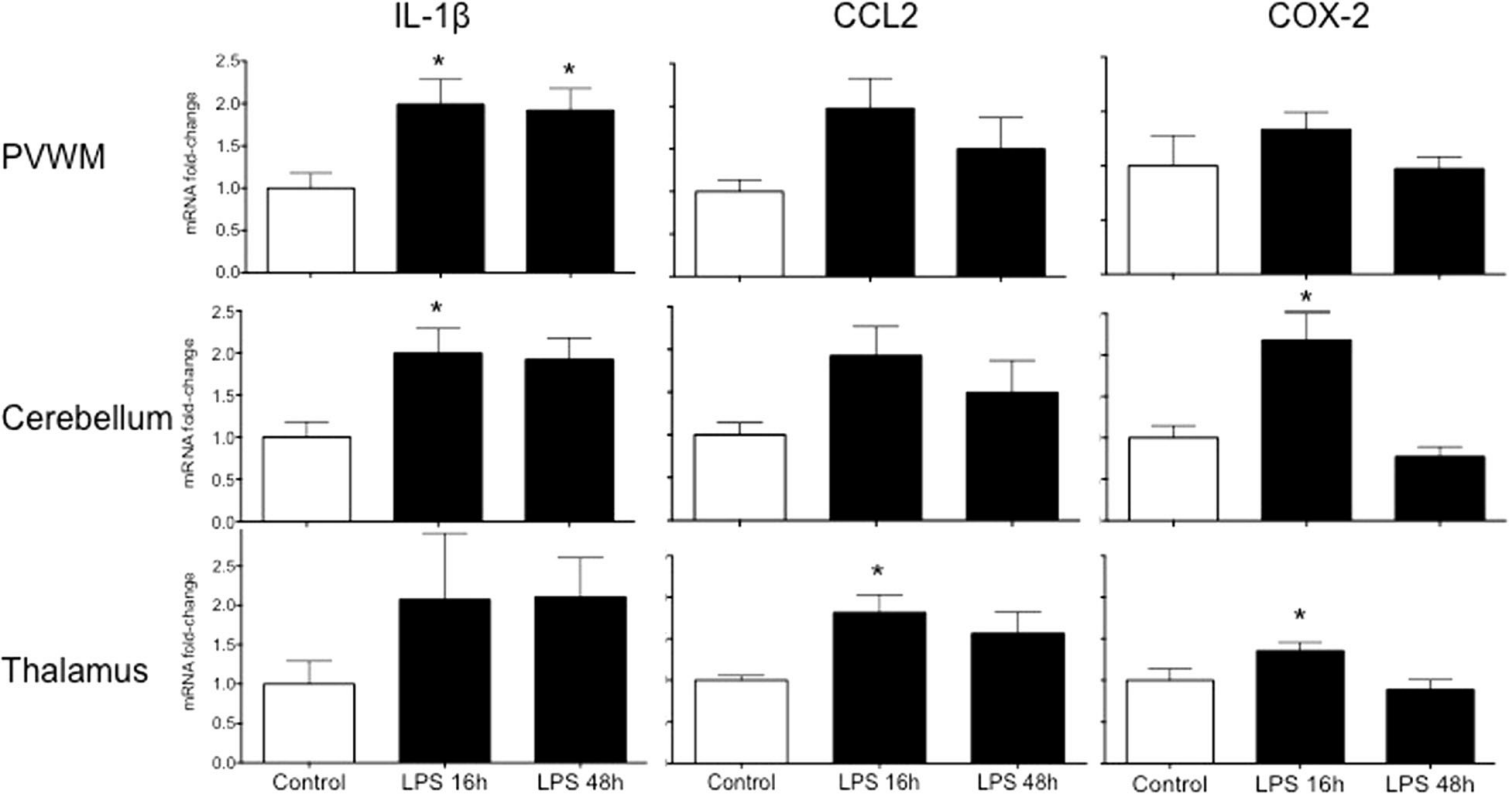
mRNA quantitation of pro-inflammatory cytokines by RT-PCR in the brain. Total mRNA was extracted from snap frozen areas of the brain. mRNA quantitation was performed by RT-PCR using rhesus specific Taqman probes. The mRNA levels are expressed as fold change relative to control after internal normalization to 18s RNA. Exposure to intra-amniotic (IA) LPS increases the expression of IL-1β in the PVWM and thalamus, COX-2 in the cerebellum and thalamus, and CCL2 in the thalamus. *p < 0.05 vs control

In the PVWM, IL-1β mRNA increased by twofold 16 and 48 h after IA LPS (*p* < 0.05) (Fig. 1). In the cerebellum, IL-1β and COX-2 mRNA expression increased 16 h after IA LPS (*p* < 0.05). In the thalamus, expression of CCL2 and COX-2 mRNA increased in the LPS 16 h group (*p* < 0.05). There were no changes in expression of IL-6, IL-10, or PTGES2 in the PVWM, cerebellum, or thalamus (Additional file 1). IL-8 mRNA expression increased in the hippocampus 16 h after IA LPS, but no other cytokines were increased. Cytokine mRNA levels were unchanged in the cortex (Additional file 2).

### IA LPS increases concentration of IL-6 concentration in the plasma and CSF

**Fig. 2:**
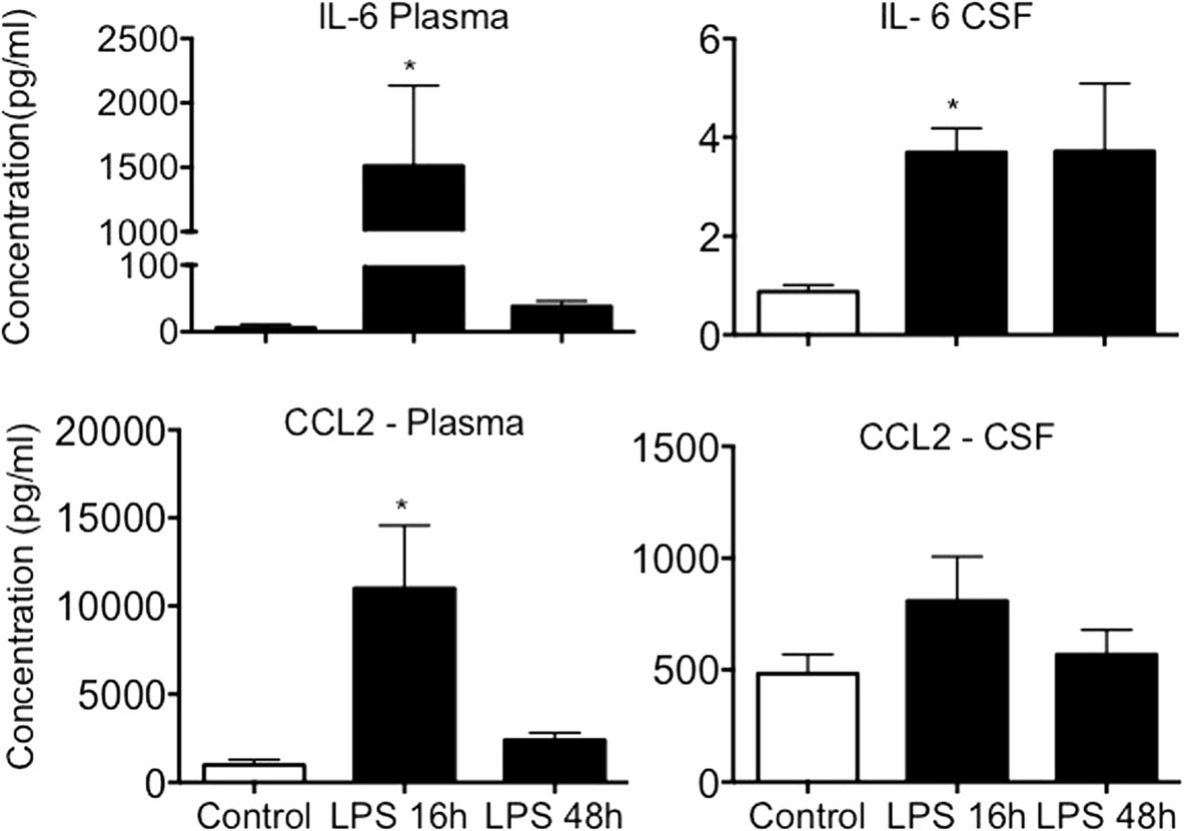
IL-6 concentration in the fetal plasma and CSF measured by ELISA. Exposure to IA LPS significantly increased the concentration of IL-6 in the plasma and CSF. *p < 0.05 vs control

IL-6 significantly increased in both the plasma and CSF after IA LPS exposure. In plasma, a 300-fold increase over controls was observed, while a more modest 4-fold increase was evident in the CSF (Fig. 2). The concentration of CCL2 was also increased in the fetal plasma at 16 h but not in the CSF 16 h after IA LPS exposure (Fig. 2). There were no differences in the levels of other cytokines including IL-1β, IL-8, IL-10, and TNF-α in the CSF or in the fetal plasma (Additional file 3).

### IA LPS induces apoptosis in the white matter

**Fig. 3:**
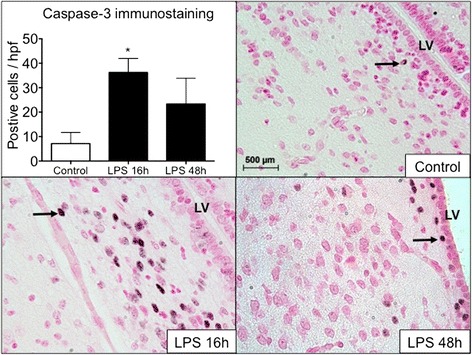
Immunohistochemical staining for activated caspase-3 in the ventricular zone. Representative sections of paraffin-embedded brain tissue from rhesus macaques fetus. Immunohistochemical staining for activated caspase-3 in the ventricular zone (*arrows*). Exposure to IA LPS increases the number of activated caspase-3 positive cells in the ventricular zone 16 h after IA LPS. Representative micrographs show the immunostaining pattern for activated caspase-3 for control, LPS 16 h, and LPS 48 h (×40). *LV* lateral ventricle. *p < 0.05 vs control

**Fig. 4:**
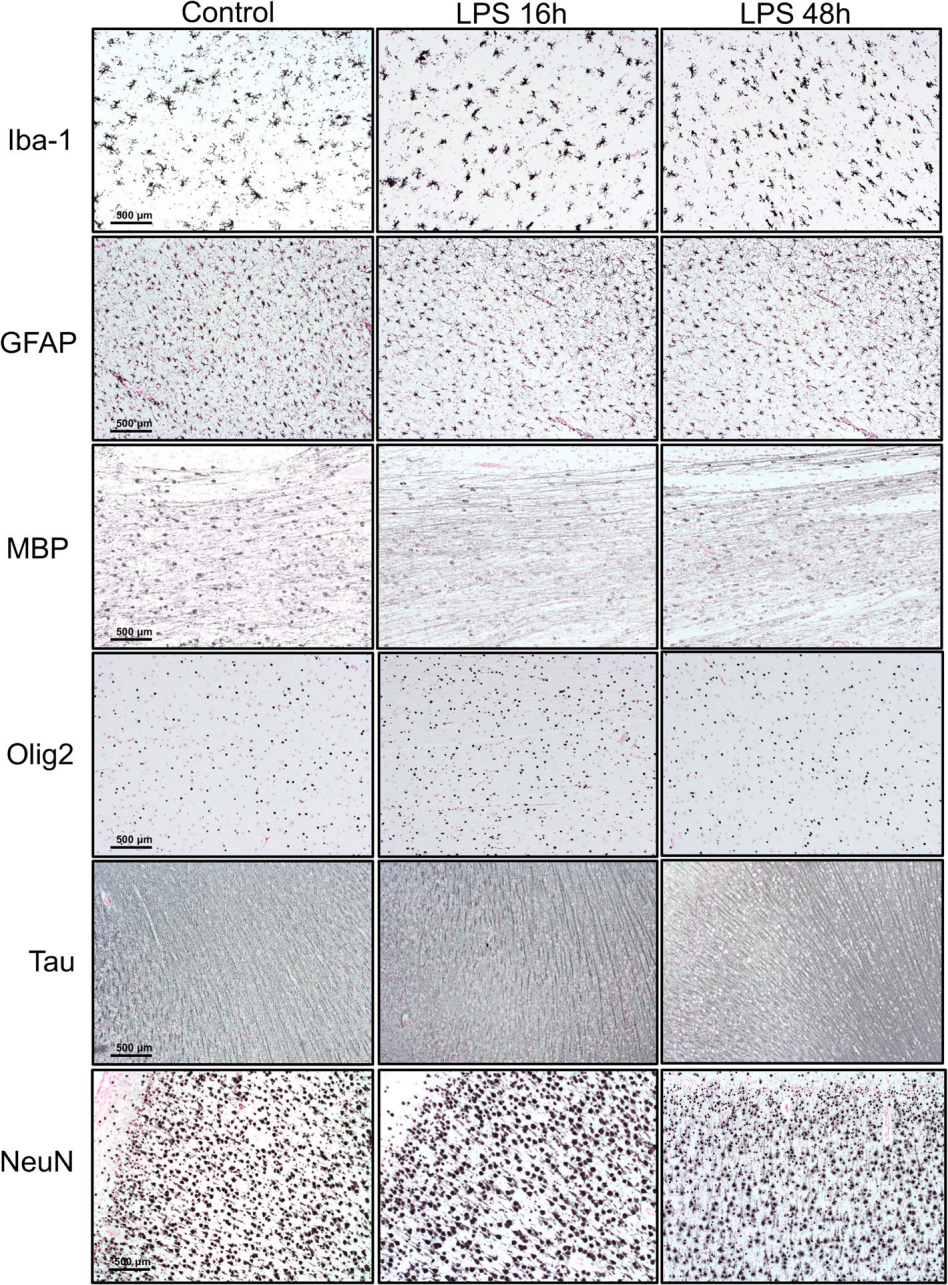
Immunostaining for cellular markers in the brain. Immunostaining for microglia (Iba-1, ×20), astrocytes (GFAP, ×10), myelin basic protein (MBP, ×10), oligodendrocytes (Olig2, ×10), axons (tau, ×10), and neurons (NeuN, ×10) was performed in coronal brain sections including for analysis of the staining pattern focused in the motor cortex, periventricular white matter, thalamus, and hippocampus. No differences were observed in staining pattern or intensity after exposure to IA LPS. *Scale bars* represent 200 μm

**Fig. 5:**
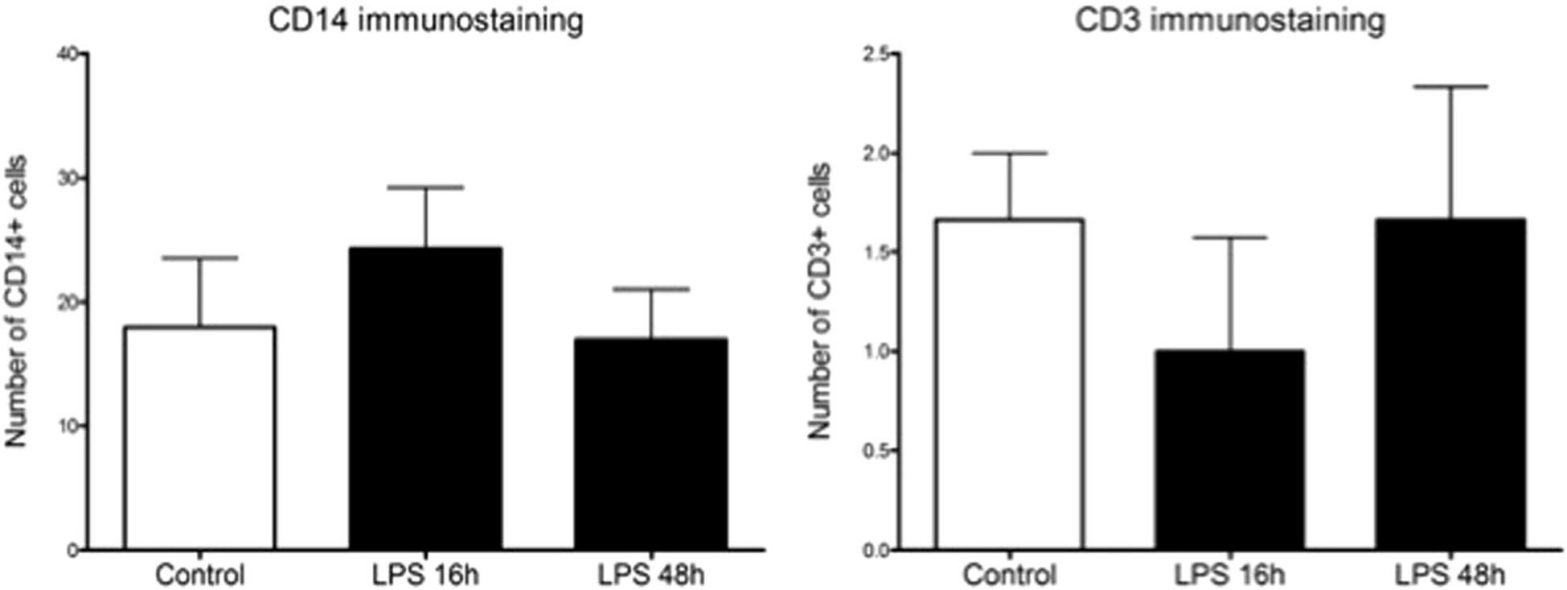
Cell count of CD14 and CD3 positive cells by immunostaining. Immunostaining was performed for CD14 and CD3 in order to investigate the presence of immune cells in the brain after IA LPS. Positive cells were counted in eight random fields per slide in one slide per animal. Numbers are reported as average total number of cells per slide. No differences were observed after exposure to IA LPS

We performed immunohistochemistry for activated caspase 3 in order to identify cells undergoing apoptosis. Caspase 3 positive cells were significantly increased in the periventricular white matter 16 h after IA LPS (*p* < 0.05), which lowered at 48 h after IA LPS (Fig. 3). There were no differences in the intensity or pattern of immunostaining for Iba-1, GFAP, MBP, Olig2, Tau, and NeuN (Fig. 4). We did not observe infiltrating leukocytes (CD3+) or infiltrating monocytes (CD14+) in the brain after IA LPS (Fig. 5).

### IA LPS induces proliferation of microglia

**Fig. 6:**
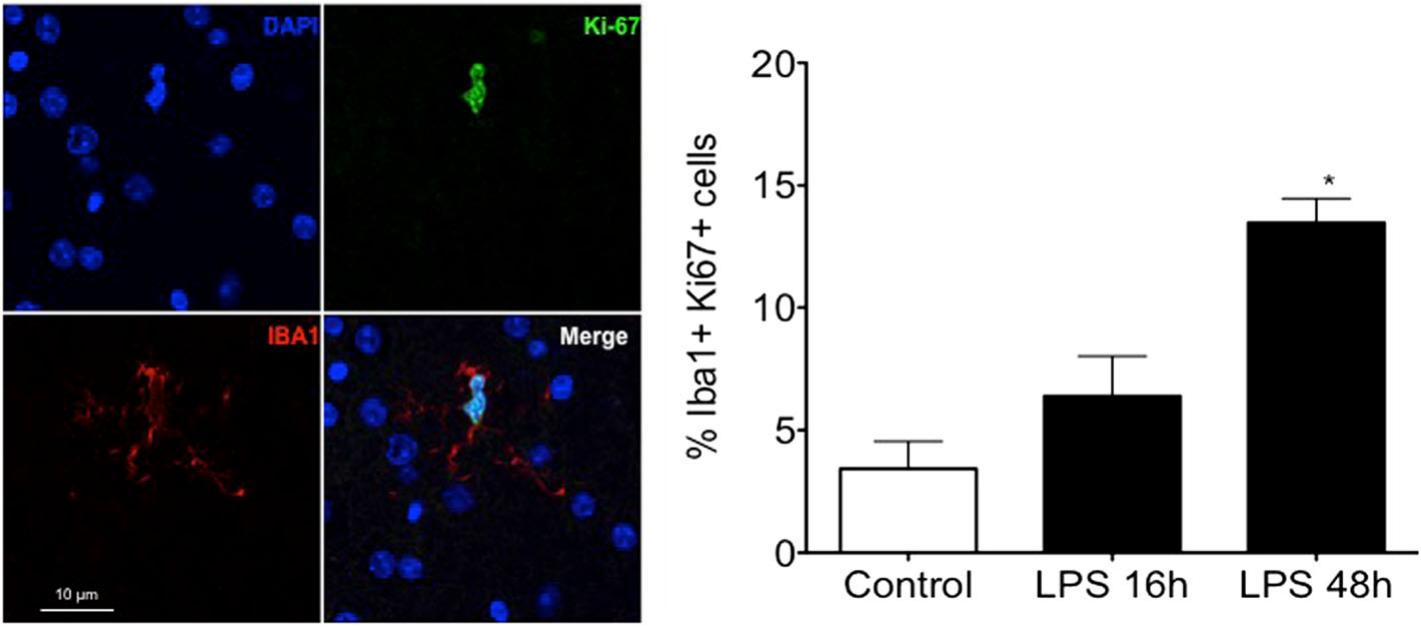
Double-labeling immunofluorescence for Iba-1 and Ki-67 and percentage of microglia labeling for Ki-67. Representative sections of paraffin-embedded brain tissue from rhesus macaques fetus. Confocal microscopy showing a microglia labeled for nuclei (*blue*), Iba-1 (*red*), and Ki-67 (green) (×60). Relative number of proliferating microglia: percentage of Iba-1positive cells that were co-stained for Ki-67. *p < 0.05 vs control

In order to identify and count the proportion of proliferating microglia in the white matter, we performed double-labeling immunofluorescence for Iba-1 and Ki-67. We observed an increased percentage of proliferating microglia 48 h after exposure to IA LPS (*p* < 0.05, Fig. 6).

## Discussion

Prematurity is the most common cause of infant mortality in the USA [23] and is frequently associated with chorioamnionitis, with at least 40 % of preterm births having prenatal inflammation [24]. Moreover, chorioamnionitis is an independent risk factor for brain injury in the neonatal period including IVH and PVL and late neurodevelopmental impairment such as CP and cognitive deficits [25–28]. Even in the absence of prematurity or signs of early neonatal brain injury such as IVH and PVL, chorioamnionitis is still associated with increased risk of CP [29]. We report the novel finding of neuro-inflammatory responses in a nonhuman primate model of chorioamnionitis induced by IA LPS. Our data suggest that neuroinflammation is a likely consequence of the fetal inflammatory response syndrome.

Even though the association between systemic inflammation and brain injury is well known, the specific mechanisms through which circulating cytokines lead to microglial activation and increased expression of cytokines in the central nervous system remain elusive [30]. We measured increased levels of IL-6 in the plasma and CSF of animals exposed to IA LPS. Increased IL-6 levels in cord blood and in the amniotic fluid have been correlated with intrauterine infection/inflammation and fetal inflammatory response syndrome [14, 31, 32]. Our data showing a significant gradient between plasma and CSF IL-6 is consistent with IL-6 crossing the blood-brain barrier through a saturable transport system [33]. Even though the plasma concentration of CCL2 was increased in LPS exposed animals, there was no significant increase in the CSF concentration of this cytokine.

The increased circulating IL-6 may cross the blood-brain barrier to the CSF. IL-6 in the central nervous system has the potential to activate the resident microglia and trigger a local inflammatory response with upregulation of pro-inflammatory cytokines. In one study of 146 preterm infants, the presence of higher CSF concentrations of IL-6, IL-10, and TNF-α was associated with white matter injury identified by magnetic resonance imaging (MRI) at term equivalent age [13]. However, there was no relationship between CSF cytokines and plasma cytokine concentrations. Elevated plasma CCL2 can also mediate the neuro-inflammatory response. CCL2, and its receptor have been implicated in the pathogenesis of brain injury due to trauma and autoimmune processes [34, 35]. Interestingly, the CCL2 levels in the CSF may be reduced in autoimmune conditions due to consumption by CCR2+ leukocytes [36]. After IA LPS, CCL2 concentration did not increase in the fetal CSF and there was no infiltration of leukocytes.

The elevation of IL-1β in the PVWM at 16 and 48 h after IA LPS may be especially important in the pathogenesis of white matter injury associated with chorioamnionitis. In preterm infants, injury to pre-myelination oligodendrocytes is central to the pathogenesis of white matter injury [11]. The expression of IL-1β and COX-2 mRNA was increased in the cerebellum 16 h after IA LPS. Although the cerebellum is not classically considered a site of brain injury in premature infants, more recent findings indicate that cerebellar underdevelopment may be common in preterm infants [37]. A strong relationship between decreased cerebellar volumes with presence of PVL has been reported [38], suggesting a common insult, such as inflammation, which is known to cause injury to the developing cerebellum [39, 40]. Another site with upregulation of pro-inflammatory cytokines after IA LPS was the thalamus, where we observed a twofold increase in CCL2 and COX-2 mRNA and a trend towards increased IL-1β mRNA. Neuropathological studies of premature infants show greater neuronal loss in the thalamus compared to the cortex and a higher incidence of thalamic injuries in the presence of PVL [41, 42]. Due to its projections to the cerebral cortex and the role of the thalamus in cognitive and social function, thalamic damage in PVL could contribute to the encephalopathy of prematurity.

COX-2 has been investigated in neonatal brain following hypoxic ischemic insults [43], but there are no data regarding a potential role of this enzyme and prostaglandins in the pathogenesis of encephalopathy of prematurity. The inducible form of COX-2 is polymorphic and the allele associated with decreased gene expression and decreased synthesis of prostaglandin is independently associated with worse long-term cognitive outcomes in preterm infants [44]. The use of the nonspecific cyclooxygenase inhibitor indomethacin in preterm newborns was associated with decreased incidence of PVL, even though indomethacin does not change long-term neurodevelopmental outcomes [45]. We found increased expression of COX-2 mRNA in the thalamus and cerebellum 16 h after exposure to IA LPS, suggesting that COX-2 may also be implicated in the brain injury induced by prenatal inflammation.

We also detected microglial proliferation after fetal exposure to IA LPS that starts at 16 h and is significant 48 h after the initial insult. Microglia are important during brain development with a variety of roles including but not limited to neuronal proliferation and differentiation, synaptic remodeling, and myelination [46]. Microglia also have a central role in the inflammatory response in the central nervous system, and PVL is associated with marked microgliosis [47]. Once activated, microglia release cytokines that can injure pre-oligodendrocyte populations [48]. It is interesting that the largest increase in microglial proliferation was after 48 h of IA LPS exposure, while cytokine mRNAs were increased at 16 h. The continued microglial proliferation could result in long-term consequences to the development of brain as has been observed in rodents models, where a single dose of intra-cerebral LPS leads to motor behavioral changes through adulthood [11].

IA LPS increased apoptosis in the periventricular white matter, as demonstrated by immunostaining for activated caspase-3. This area is a frequent site of injury in preterm infants [3]. Our findings are consistent with other models of preterm and neonatal brain injury. In rodents, intra-cerebral LPS induces apoptosis in the white matter [49]. In the sheep model, IA LPS increases the number of apoptotic cells in the PVWM and cortical area, with associated microglial activation [20, 50]. In this model, longer exposure to IA LPS resulted in increased injury and abnormalities on EEG with increased delta wave frequency after 14 days [50]. The association of IA LPS, early expression of inflammatory markers in the brain, and late functional changes supports the idea that exposure to prenatal inflammation causes specific inflammatory responses in the brain and may result in the adverse neurodevelopmental outcomes observed in preterm newborns exposed to chorioamnionitis.

## Conclusions

IA injection of LPS in a nonhuman primate results in modest acute inflammatory responses in the central nervous system evidenced by increased cytokine in the CSF and cytokine mRNA levels and microglial proliferation in the brain. The inflammatory response is associated with apoptosis in the periventricular white matter. This novel model of fetal brain injury induced by chorioamnionitic inflammation should be useful for understanding the pathogenesis of white matter injury associated with prenatal inflammation. Our results suggest that the brain injury in fetuses exposed to chorioamnionitis could be mediated by circulating IL-6 and/or CCL2.

## Additional files

Additional file 1: mRNA quantitation of IL-6, IL-10, and PTGES2 in the PVWM, cerebellum, and thalamus. (DOCX 69 kb) Additional file 2: mRNA quantitation of pro-inflammatory cytokines in the hippocampus and cortex. (DOCX 14 kb) Additional file 3: Cytokine concentrations in the fetal plasma and fetal cerebrospinal fluid (CSF) measured by ELISA. (DOCX 54 kb)

## Abbreviations

CCL2: Chemokine ligand 2
CD: Cluster differentiation
CNPRC: California National Primate Research Center
CSF: Cerebrospinal fluid
COX-2: Cyclooxygenase-2
CP: Cerebral palsy
ELISA: Enzyme-linked immunosorbent assay
GFAP: Glial fibrillary acidic protein
Iba1: Ionized calcium-binding adaptor molecule 1
IA: Intra-amniotic
IL: Interleukin
Ki-67: Marker of proliferation
MBP: Myelin basic protein
MRI: Magnetic resonance imaging
NeuN: Neuronal nuclei
Olig2: Oligodendrocyte transcription factor 2
LPS: Lipopolysaccharide
PBS: Phosphate buffer saline
PTGES2: Prostaglandin E synthase 2
PVL: Periventricular leukomalacia
PVWM: Periventricular white matter
TNF: Tumor necrosis factor
WBC: White blood cell count
