# Supplementary material for: Intra-amniotic LPS causes acute neuroinflammation in preterm rhesus macaques
Source: J Neuroinflammation. 2016 Sep 6;13(1):238. doi: 10.1186/s12974-016-0706-4 (PMC5011884; doi:10.1186/s12974-016-0706-4)
Supplement: Additional file 1: — mRNA quantitation of IL-6, IL-10, and PTGES2 in the PVWM, cerebellum, and thalamus. (DOCX 69 kb) [file 12974_2016_706_MOESM1_ESM.docx]

| Brain area | Group | IL-6 | IL-10 | PTGES2 |
| --- | --- | --- | --- | --- |
| PVWM | Control | 1.0 ± 0.24 | 1.0 ± 0.41 | 1.0 ± 0.33 |
|  | 16h | 1.5 ± 2.12 | 1.1 ± 0.65 | 0.7 ± 0.26 |
|  | 48h | 0.9 ± 0.53 | 1.0 ± 0.41 | 0.8 ± 0.39 |
| Cerebellum | Control | 1.0 ± 1.2 | 1.0 ± 0.64 | 1.0 ± 0.24 |
|  | 16h | 1.0 ± 0.64 | 0.9 ± 0.63 | 1.1 ± 0.31 |
|  | 48h | 0.4 ± 0.26 | 0.9 ± 0.37 | 0.9 ± 0.40 |
| Thalamus | Control | 1.0 ± 1.02 | 1.0 ± 0.45 | 1.0 ± 0.20 |
|  | 16h | 0.8 ± 0.55 | 0.6 ± 0.10 | 1.0 ± 0.47 |
|  | 48h | 0.7 ± 0.28 | 1.4 ±0.73 | 1.1 ± 0.11 |

*Additional file 1.* mRNA quantitation of Il-6, IL-10, and PTGES2 in the PVWM, cerebellum, and thalamus.

*Legend.* Total mRNA was extracted from snap frozen areas of the brain. mRNA quantitation was performed by RT-PCR using rhesus specific Taqman probes. The mRNA levels are expressed as fold change relative to control after internal normalization to 18s RNA. There were no differences in the mRNA expression of IL-6, IL-10, and PTGES2 in the PVWM, cerebellum, or thalamus.
