# Supplementary material for: Intra-amniotic LPS causes acute neuroinflammation in preterm rhesus macaques
Source: J Neuroinflammation. 2016 Sep 6;13(1):238. doi: 10.1186/s12974-016-0706-4 (PMC5011884; doi:10.1186/s12974-016-0706-4)
Supplement: Additional file 2: — mRNA quantitation of pro-inflammatory cytokines in the hippocampus and cortex. (DOCX 14 kb) [file 12974_2016_706_MOESM2_ESM.docx]

| Brain area | Group | IL-1β | CCL2 | TNF-α | IL-6 | IL-8 | IL-10 | COX-2 | PTGES2 |
| --- | --- | --- | --- | --- | --- | --- | --- | --- | --- |
| Hippocampus | Control | 1.0 ± 0.39 | 1.0 ± 0.54 | 1.0 ± 0.84 | 1.0 ± 0.48 | 1.0 ± 0.23 | 1.0 ± 0.38 | 1.0 ± 0.62 | 1.0 ± 0.43 |
|  | 16h | 1.6 ± 0.49 | 1.3 ± 0.63 | 0.9 ± 0.42 | 0.9 ± 0.43 | 4.4 ± 1.86* | 0.9 ± 0.36 | 1.0 ± 0.84 | 0.8 ± 0.14 |
|  | 48h | 1.7 ± 0.80 | 1.6 ± 1.13 | 0.7 ± 0.21 | 0.7 ± 0.24 | 3.8 ± 2.50 | 1.2 ± 0.09 | 0.8 ± 0.70 | 0.8 ± 0.16 |
| Cortex | Control | 1.0 ± 0.68 | 1.0 ± 0.45 | 1.0 ± 0.78 | 1.0 ± 0.32 | 1.0 ± 0.57 | 1.0 ± 0.42 | 1.0 ± 0.36 | 1.0 ± 0.96 |
|  | 16h | 1.2 ± 1.09 | 0.8 ± 0.44 | 0.6 ± 0.37 | 0.8 ± 0.55 | 0.9 ± 0.37 | 0.85 ± 0.35 | 1.0 ± 0.34 | 0.9 ± 0.76 |
|  | 48h | 1.15 ± 0.79 | 1.2 ± 0.53 | 1.0 ± 0.52 | 0.54 ± 0.05 | 1.25 ± 0.67 | 1.6 ± 0.52 | 0.6 ± 0.27 | 1.2 ± 0.92 |

*Additional file 2.* mRNA quantitation of pro-inflammatory cytokines in the hippocampus and cortex.

*Legend.* Total mRNA was extracted from snap frozen areas of the brain. mRNA quantitation was performed by RT-PCR using rhesus specific Taqman probes. The mRNA levels are expressed as fold change relative to control after internal normalization to 18s RNA. IL-8 mRNA increased 16 hours after LPS exposure in the hippocampus. *p<0.05
