# Supplementary material for: Intra-amniotic LPS causes acute neuroinflammation in preterm rhesus macaques
Source: J Neuroinflammation. 2016 Sep 6;13(1):238. doi: 10.1186/s12974-016-0706-4 (PMC5011884; doi:10.1186/s12974-016-0706-4)
Supplement: Additional file 3: — Cytokine concentrations in the fetal plasma and fetal cerebrospinal fluid (CSF) measured by ELISA. (DOCX 54 kb) [file 12974_2016_706_MOESM3_ESM.docx]

Additional file 3. Cytokine concentrations in the fetal plasma and fetal cerebrospinal fluid (CSF) measured by ELISA.

|  |  | IL-1β | TNF-α | IL-8 | IL-10 |
| --- | --- | --- | --- | --- | --- |
| Plasma | Control | 0.14 ± 0.19 | 25 ± 41.8 | 940 ± 733 | 24 ± 40 |
|  | LPS 16h | 8.01 ± 9.2 | 20 ± 7.7 | 1637 ± 866 | 112 ± 88 |
|  | LPS 48h | 0.17 ± 0.33 | 9.5 ± 3.7 | 720 ± 466 | 18 ± 12 |
| CSF | Control | <0.01 | <0.01 | 31.7 ± 14 | <0.01 |
|  | LPS 16h | <0.01 | <0.01 | 27 ± 7 | <0.01 |
|  | LPS 48h | <0.01 | <0.01 | 91 ± 9 | <0.01 |

*Legend.* Cytokine concentrations were measured by ELISA in the fetal plasma and fetal CSF. There was no difference in the concentration of IL-1β, TNF-α, IL-8, or IL10.
